# Heat Treatments for Stress Relieving AlSi9Cu3 Alloy Produced by Laser Powder Bed Fusion

**DOI:** 10.3390/ma14154184

**Published:** 2021-07-27

**Authors:** Jacopo Fiocchi, Chiara Colombo, Laura Maria Vergani, Alberto Fabrizi, Giulio Timelli, Ausonio Tuissi, Carlo Alberto Biffi

**Affiliations:** 1 CNR ICMATE, National Research Council, Institute of Condensed Matter Chemistry and Technologies for Energy, Unit of Lecco, CNR ICMATE, via Previati 1/e, 23900 Lecco, Italy; jacopo.fiocchi@icmate.cnr.it (J.F.); ausonio.tuissi@cnr.it (A.T.); 2 Department of Mechanical Engineering, Politecnico di Milano, via La Masa 1, 20156 Milano, Italy; chiara.colombo@polimi.it (C.C.); laura.vergani@polimi.it (L.M.V.); 3 Department of Engineering and Management, University of Padua, Stradella San Nicola 3, 36100 Vicenza, Italy; fabrizi@gest.unipd.it (A.F.); giulio.timelli@unipd.it (G.T.)

**Keywords:** laser powder bed fusion, selective laser melting, Al alloys, AlSi9Cu3, heat treatment, microstructure, residual stresses

## Abstract

The present work explores the effect of a stress relieving heat treatment on the microstructure, tensile properties and residual stresses of the laser powder bed fused AlSi9Cu3 alloy. In fact, the rapid cooling rates together with subsequent heating/cooling cycles occurred during layer by layer additive manufacturing production make low temperature heat treatments desirable for promoting stress relaxation as well as limited grain growth: this combination can offer the opportunity of obtaining the best compromise between high strength, good elongation to failure and limited residual stresses. The microstructural features were analysed, revealing that the high cooling rate, induced by the process, caused a large supersaturation of the aluminum matrix and the refinement of the eutectic structure. Microhardness versus time curve, performed at 250 °C, allowed to identify a stabilization of the mechanical property at a duration of 25 h. The microstructure and the mechanical properties of the samples heat treated at 25 h and at 64 h, considered as a reference for the conventionally produced alloy, were compared with the ones of the as-built alloy. Finally, it was shown that a 59% reduction of the principal residual stresses could be achieved after the 25 h-long treatment and such evolution was correlated to the mechanical behaviour.

## 1. Introduction

Laser powder bed fusion (LPBF) of aluminium alloys has witnessed a promising growth in the last few years and can now be considered, in a way, a well-established industrial reality. However, few alloys have driven considerable attention: among these the well-known AlSi10Mg alloy has been widely studied and its properties are now relatively deeply assessed under many different perspectives, including processability [1,2], microstructure [3], static [4] and dynamic [5] mechanical properties and fatigue life [6,7]. Moreover, it has been widely demonstrated that LPBF-built aluminum parts require post-processing heat treatments [8] in order to remove residual stresses [9] and, potentially, homogenize the microstructure and the mechanical behaviour [10]. In this respect, owing to the arising fine microstructure and large supersaturation, it has been recognized by numerous authors that temperatures and durations of the aforementioned treatments need to be properly tuned according to the specific features of the LPBF-ed alloys [11,12].

Therefore, it appears now of great interest to deepen the knowledge of other alloys, when processed by LPBF. Researchers have pursued different strategies, including developing new alloys specifically dedicated to additive manufacturing processes [13] and adapting known compositions to selective laser melting [14,15]. Another more conservative approach consists of synthetizing by LPBF alloys, which are currently widely produced by conventional routes and are already employed in specific industrial applications. This last approach aims at obtaining two kinds of advantages: first, the higher design flexibility granted by LPBF may yield more effective final components [16] (e.g., by incorporating multiple functionalities or allowing weight savings); secondly, the material’s properties may be strongly improved thanks to the microstructural refinement induced by the high cooling speeds involved in the process [17]. In this light, the AlSi9Cu3 alloy appears to be an interesting candidate for application in additive manufacturing. In fact, its high Si content makes LPBF processability likely feasible [18], it is widely employed in the automotive sector [19] (usually produced by high pressure die casting, HPDC) and the presence of Cu makes it age-hardenable [20,21].

Some of the authors of the present work have recently explored the LPBF processability of a primary AlSi9Cu3 alloy [22], whose microstructure had also been previously analysed in details by Fousova et al. [23]. It was demonstrated that, due to the refined microstructure and the low density of defects, the LPBFed alloy could outperform the mechanical behaviour of the conventional AlSi9Cu3 alloy produced by HPDC. In [24] microstructural evolution of the LPBFed alloy upon direct ageing (140 °C–180 °C) was studied by transmission electron microscopy and compared to the one induced by conventional T6 treatment. It was shown that strengthening was mainly due to high-density θ’’ precipitation in solution treated samples, whereas a combination of semi-coherent θ’ and Si-rich precipitates was found across the Al matrix of directly aged parts. A further type of thermal treatment, which is commonly applied to LPBFed Al alloys, is stress relieving, usually obtained by annealing in the 200 °C–350 °C range. This treatment aims at removing residual stresses, which derive from volumetric changes of the material upon cooling and are exacerbated by the layer-by-layer nature of LPBF [25,26]. Indeed, residual stresses are known to negatively affect the behaviour of LPBFed aluminium alloys under several perspectives, including fatigue life [27] and corrosion resistance [28]. Their reduction or removal is usually obtained by means of annealing treatments, which are performed in a temperature range, which lies between the one used for ageing treatment (usually from 140 °C to 180 °C) and the one able to induce a proper solubilization of the material (above 480 °C). Inside this temperature interval, two different ranges can be identified, which differently affect the microstructure of LPBFed Al-Si alloys [29]: the lower one (up to 270 °C) is able to reduce residual stresses without affecting the eutectic cellular Si network; the upper one on the contrary also induces the spheroisation of the Si network, leading to a sharp decrease in mechanical resistance [9].

Therefore, considering the already available results, the present work aims at studying the influence of a stress relieving treatment on the microstructure and mechanical properties of an LPBFed AlSi9Cu3 alloy. A treatment temperature meant to remove residual stress while not affecting the morphology of the eutectic Si network was chosen, so as to avoid an excessive reduction of mechanical resistance with respect to as-built parts. The effect of the selected annealing temperature on the evolution of microstructure, residual stresses and mechanical behaviour was explored.

## 2. Materials and Methods

The primary AlSi9Cu3 (wt.%) samples were produced from gas-atomized powder (LPW Technology, Ltd, Philadelphia, PA, USA), which exhibited a size range of 20–63 µm and the composition reported in Table 1. A Renishaw AM400 machine (Renishaw, Wotton-under-Edge, UK), equipped with a 400 W pulsed wave fibre laser, was used to build two types of samples: rectangular bars for microstructural analysis (50 × 10 × 10 mm^3^), built with their major axis parallel to the building plate, and thin laminas (70 × 2 × 20 mm^3^), lying in the sagittal plane, for tensile testing. Employed processing parameters, whose optimization is reported in a previous work [22], are summarized in Table 2. A standard meander scanning strategy was used and each scanning layer was rotated by 67° with respect to the previous one.

In order to investigate the material response to a stress relieving treatment, annealing oneat 250 °C, followed by air cooling, was performed for times ranging from 5 min to 64 h.

**Figure 1:**
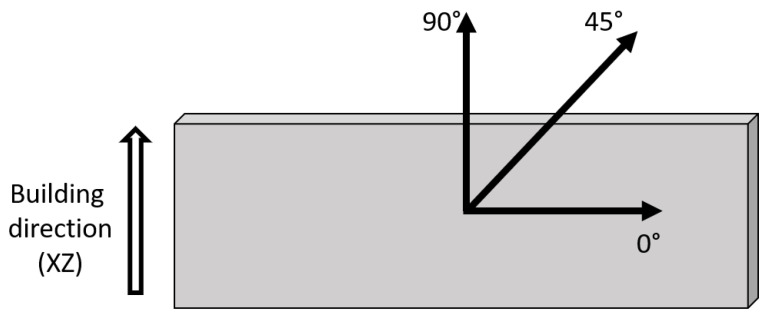
Schematic depicting the reference directions used during residual stress measurements.

Different experimental techniques were used in order to follow the evolution of material properties upon annealing. Vickers microhardness was measured on samples annealed for different times by means of a Leitz durometer (Future-Tech Corp FM-700, Tokyo, Japan ), applying a 200 gf load for 15 s. Tensile tests were performed on samples treated for selected durations according to the E8/E8M-11 ASTM standard on sub-sized dog-bone specimens with a crosshead speed of 0.5 mm/min (3.3 × 10^−4^ s^−1^) using an MTS 2M testing machine (MTS Systems Corporation, Eden Prairie, MN, USA), equipped with an extensometer, at room temperature. At least three samples per condition were tested. Residual stresses were measured on seldected specimens with an X-ray diffractometer X-Stress 3000 (Stresstech Oy, Vaajakoski, Finland). The measurements were performed by the sin2ψ method, with the following setup parameters: collimator with 3 mm diameter, Cr Kα X-ray tube with voltage 30 kV and current 6.5 mA, 7 tilt angles equally spaced between −45° and +45, and exposure time 30 s. As depicted in Figure 1, the measurements were performed at the surface, given the small thickness of the samples, along three in-plane reference directions: 0°, i.e., parallel to the building plate (XY); 45°; and 90°, i.e., along the building direction (XZ).

The effect of annealing time on the microstructure was investigated by using a field emission gun scanning electron microscope (FEG-SEM, Quanta 250, FEI, Thermo Fisher Scientific, 5350 NE Dawson Creek Drive, Hillsboro, OR, USA) operating at 20 kV equipped with an energy dispersive X-ray spectrometer (EDS, EDAX) and an electron back-scattered diffraction (EBSD, EDAX) detector; in-depth microstructural observations were performed by a transmission electron microscope (TEM, JEOL, Thermo Fisher Scientific, 5350 NE Dawson Creek Drive, Hillsboro, OR, USA) working at 200 kV.

All microstructural analyses of as-built and annealed materials were carried out on the orthogonal plane to the building direction (XY-section); prior to the EBSD analysis, the sections were finely polished with silica colloidal suspension with a 0.04 µm-particle size; the EBSD scans were conducted over an area of about 500 × 500 µm^2^ with a step size of 1 µm. Then, the samples were etched with Keller’s reagent for SEM observations. TEM investigations were conducted on 3-mm discs thinned up to electron transparency by using a precision ion polishing system (PIPS, GATAN, Las Positas Blvd. Pleasanton, CA, USA). 

## 3. Results and Discussion

**Figure 2:**
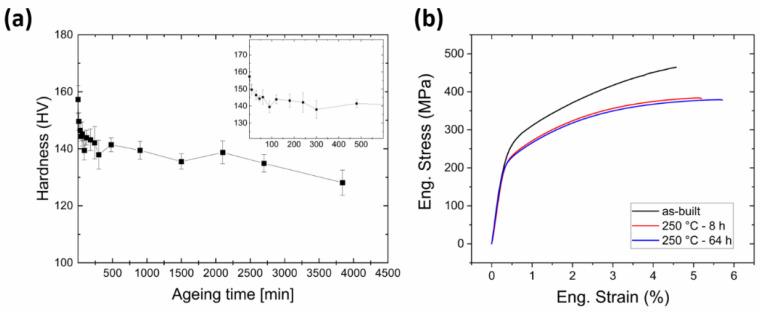
(**a**) evolution of hardness upon isothermal annealing at 250 °C; (**b**) tensile curves of as-built and annealed samples.

In order to identify the correct time frame for stress relieving treatment of LPBFed AlSi9Cu3 parts, as-built samples were subjected to annealing at 250 °C for various durations. The resulting evolution of hardness was recorded and is shown in Figure 2a. As common in LPBFed Al-Si alloys, annealing in an intermediate temperature range, roughly comprised between 240 °C and 290 °C [30,31], induces a rather sharp decrease of hardness for relatively short treatment times (from 157.3 HV in as-built condition to 143.9 HV after 2 h), and an almost flat plateau for longer treatment duration. Smoother hardness decay is expected in this second stage but the magnitude of such decrement is largely inferior to the one experienced by the alloy for a short annealing time. In fact, 8 h (480 min) was chosen as a reference time for full stabilization in the present work: further characterizations will address as-built samples and parts treated at 250 °C for 8 h (141.4 HV) and 64 h (128.1 HV). The decrement of hardness between as-built and stabilized samples amounts to 10.1%, which is somehow smaller than the one revealed in the well-known AlSi10Mg alloy treated at a similar temperature (244 °C, 23.9% decrement) [9]. On the contrary, the time-dependency of the trends in the two alloys appears extremely similar, suggesting that the main underlying process might still be the relaxation of residual stresses. The tensile behaviour associated with the above-mentioned conditions is shown in Figure 2b. It is immediately evident that the stress relieving treatment induced a decrease of both yield strength (from 266.1 ± 0.1 MPa in as-built condition to 231.8 ± 1.5 MPa and 229.5 ± 1.1 MPa after 8 h and 64 h annealing, respectively) and ultimate tensile strength (from 462.5 ± 0.2 MPa in as-built condition to 380.5 ± 1.2 MPa and 380.3 ± 0.7 MPa after 8 h and 64 h annealing, respectively). On the contrary, and accordingly with what could be expected, elongation to failure showed a continuous improvement as treatment time increased (from 4.50% ± 0.12 in as-built condition to 4.82% ± 0.51 and 5.77% ± 0.14 after 8 h and 64 h annealing, respectively). It is remarkable that all the considered samples, even after prolonged stress relieving, display mechanical properties higher than the ones required by the En 1706:1998 standard, which requires 140 MPa as yield strength, 240 MPa as ultimate tensile strength and 1% as elongation to failure.

**Figure 3:**
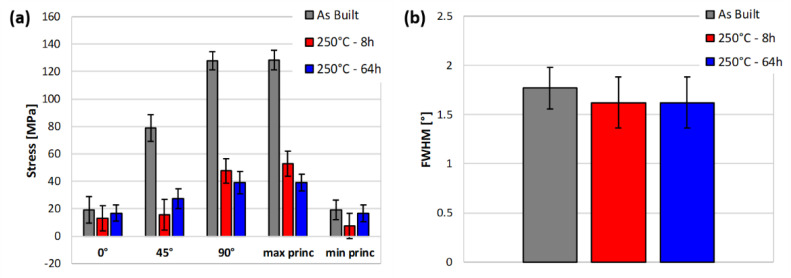
Results of the XRD measurements at the surface of the specimens: (**a**) residual stresses in the three directions and resulting maximum and minimum principal stresses; (**b**) full width at half maximum (FWHM) parameter.

Figure 3a shows the residual stresses measured on samples in different annealing conditions in the three reference directions (0°−45°−90°) and the corresponding principal stresses. All residual stresses were found to be of tensile nature at the surface of the samples: therefore, their reduction or removal is of paramount importance, as they are expected to negatively affect fatigue resistance of LPBFed parts [7]. The maximum tensile principal stress occurs for all the samples at the 90° direction, i.e., along the building direction. It is evident from this histogram that the as-built specimen experiences residual stresses higher than the two thermally treated. Considering the maximum principal stress, indeed, the stress reduction with respect to the as-built specimen is 59% and 70% for the specimens treated at 250 °C for 8 h and 64 h, respectively. The two thermal treatments have similar effects on the residual stresses, with a difference of 14 MPa in the average value of the maximum principal stress and slightly overlapped confidence bounds. These results further confirm that most of the changes induced by heat treatment take place during its initial 8 h: therefore, the 8 h-long treatment appears as an optimized methodology, reaching a sort of plateau effect in terms of mechanical performance and residual stresses. In other words, increasing the treatment time would not induce a net beneficial effect.

Besides, Figure 3b shows the Full Width at Half Maximum (FWHM) of the diffraction peaks, which is a parameter also obtained from the X-ray measurements. The FWHM parameter has been typically studied and related to grain distortion, dislocation density and type II micro residual stresses [32]. High values of this parameter are an index of the material hardening, and, for this reason, it is typically used to analyse the effect of mechanical and thermal treatments, as in [6,33]. In the present study, the two thermally treated specimens exhibit the same value, while a slightly higher value (8% on average) is detected for the as-built specimen. In particular, in [6] it was shown that, for a similar LPBFed aluminium alloy, the FWHM slightly decreases with heat treatments; this observation is consistent with the result of Figure 3b. The decrease of the FWHM parameter, i.e., the narrowing of diffraction peaks, reflects the hardness reduction underwent by the material after the heat treatment (see Figure 2a) and furtherly supports the evolution of mechanical properties upon heat treatment.

**Figure 4:**
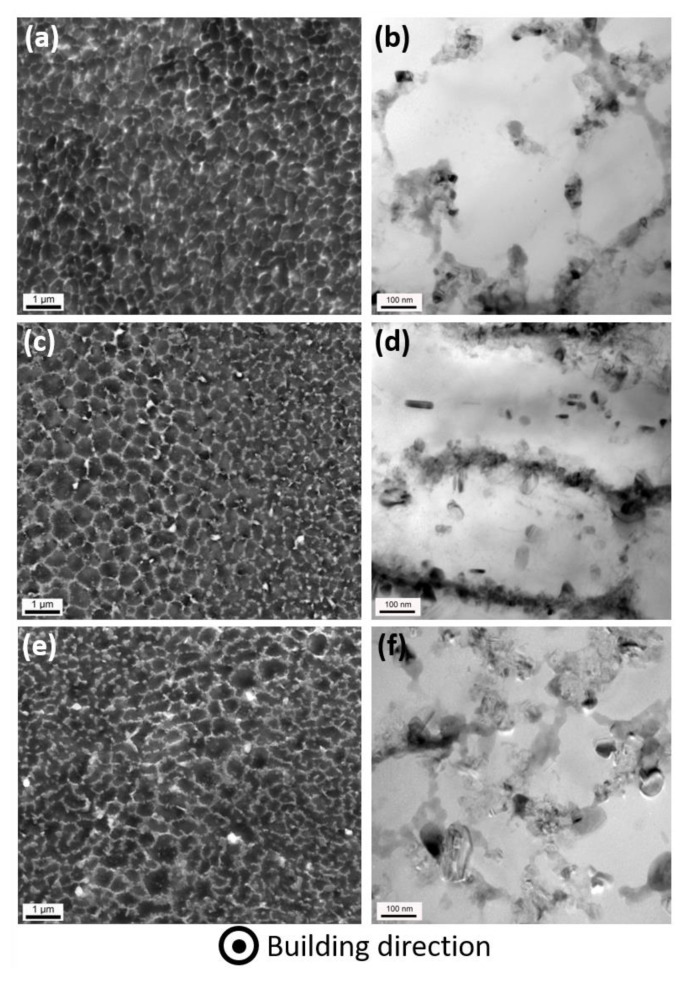
(**a**,**c**,**e**) SEM and (**b**,**d**,**f**) TEM micrographs of (**a**,**b**) as-built and annealed samples for (**c**,**d**) 8 h and (**e**,**f**) 64 h. All micrographs depict the XY section of the considered samples.

In order to better understand the origin and evolution of the alloy’s mechanical properties, microstructural analyses were performed on multiple scales. The microstructure of the as-built material displays the typical cellular Al–Si eutectic structure, which also characterises the more common LPBFed AlSi10Mg alloy [34]. The Si-rich eutectic network (light grey areas in Figure 4a) appears as almost continuous at this length scale and no trace of other second phases can be found. Indeed, from the bright-field TEM micrograph at higher magnification (Figure 4b), it may be appreciated that the eutectic Si network is actually composed of distinct extremely fine Si particles, which surround primary α-Al cells in the 500–700 nm range. This structure has been reported to be responsible for the extremely high work hardening rate characterizing as-built LPBFed Al-Si alloys [4]. After annealing at 250 °C for 8 h the alloy’s microstructure is still dominated by the presence of the eutectic Si network which, at relatively low magnification (Figure 4c) does not present any evident changes. On the contrary, an evident discontinuity is represented by the appearance of several quite large and bright particles, apparently randomly distributed across the matrix. Such particles were found to be rich in Cu, with a composition close to the stoichiometric Al_2_Cu one. Moreover, at higher magnification, Si nanoparticles with a size up to about 50 nm are found to precipitate inside the α-Al matrix (Figure 4d).

By further extending the annealing treatment, the continuous Si-rich eutectic network starts to fragment although the cellular microstructure seems to be still recognizable (Figure 4e); in addition, a higher density of large particles is observed. Beside the Si particles, EDS elemental distribution clearly revealed that brighter particles are indeed Cu-rich phases, as also reported by Roudnická et al. for a thermal treated AlSi9Cu3Fe alloy [24]. TEM micrograph at higher magnification displays more distinctly the coarsening of Si particles as the thermal annealing is extended up to 64 h (Figure 4f): the intercellular network, partially preserved, now mainly consists of coarse globular particles even larger than 100 nm; moreover, as a consequence of Oswald ripening, the fraction of finer precipitates within the α-Al cells decreases. Such modifications of the microstructure well fit with the evolution of mechanical properties: indeed, stress relieved samples displayed reduced strength as a consequence of the reduced fraction of fine Si precipitates, and slightly improved ductility. Moreover, annealed samples appear to be characterized by lower strain-hardening ability, which may be due to the coarsening of particles composing cell boundary and is also related to the ability to accommodate larger deformation before failure.

**Figure 5:**
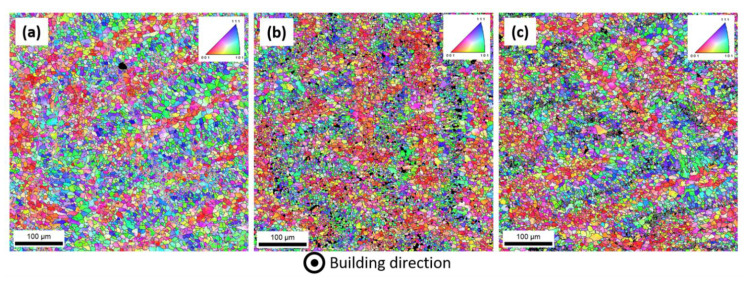
EBSD orientation maps on XY section of (**a**) as-built and annealed samples for (**b**) 8 h and (**c**) 64 h; in the inset, the orientation colour code for cubic crystal.

EBSD orientation maps in Figure 5 display the morphology and size of grains for as-built and annealed samples for 8 h and 64 h: in general, larger equiaxial grains form in the core of melt pools with preferential orientation along the (001) direction parallel to the building direction (XZ), whereas finer grains appear around the melt pool boundaries, exhibiting orientations between the [101] and [111] directions. This is consistent with other works [35,36], which have also shown that the grain size of the similar LPBFed AlSi10Mg alloy is scarcely influenced by thermal treatments performed at relatively low temperature. In fact, the average equivalent diameters of the grains (Table 3) for the three conditions were found to be essentially unchanged and, furthermore, the overlapping confidence bands indicate that no statistically significant variation could be observed. Therefore, it can be safely stated the annealing treatment has not a significant influence on the grain size of the alloy. In this respect, the retention of such a fine grain size, as well as the one of a relatively intact Si network, play a fundamental role in maintaining a satisfactory strength of the alloy, even after prolonged exposure to elevated temperature, as indicated by the reported mechanical results.

## 4. Conclusions

The present paper explored the possibility of tailoring the microstructural and mechanical behaviour of LPBFed AlSi9Cu3 alloy through dedicated heat treatments. Annealing at intermediate temperature (250 °C) was shown to greatly reduce the residual stresses, building up in LPBFed parts during the process itself. In particular, the 8 h-long treatment can be considered an optimized condition, as it induced a 56% reduction of maximum principal residual stress with respect to the as-built condition and, concurrently, allowed the retaining of satisfactory mechanical strength. Microstructural evolution during thermal treatments was studied as well: annealing at 250 °C was shown to mainly affect the size and distribution of precipitates lying in the interior of α-Al cells during initial stages; on the contrary, prolonged exposure to relatively high temperature induce a partial coarsening of eutectic Si particles at cell boundaries and, concurrently, the dissolution of finer precipitates inside the cells. As a consequence, a limited reduction of mechanical strength and, conversely, a limited increase in ductility were obtained after the 64 h-long treatment.

In summary, the 8 h-long treatment at 250 °C was found to constitute a valid procedure for obtaining effective stress relieving of LBFed AlSi9Cu3 parts, while maintaining satisfactory mechanical properties.

## Figures and Tables

**Table 1:** Chemical composition of the utilized powder, as measured by ICP.

	Al	Si	Cu	Fe	Mg	Ti
Wt.%	Bal.	8.92	2.367	0.339	0.028	0.007
Std dev.		0.625	0.106	0.001	0.009	0.0005

**Table 2:** Used LPBF processing parameters.

Parameters	Values
Exposure time (µs)	40
Power (W)	275
Hatch distance (µm)	90
Point distance (µm)	90
Laser spot size (µm)	65
Atmosphere	Ar
Thickness layer (µm)	30
Platform temperature (°C)	30

**Table 3:** Average grain size obtained from EBSD data for as-built and 250°C-heat treated samples for 8 and 64 h.

	As-Built	250 °C-8 h	250 °C-64 h
Mean Grain Size (µm)	4.8	4.6	4.3
Std dev.	2.3	2.0	2.2

## Data Availability

The raw/processed data required to reproduce these findings cannot be shared at this time as the data also form part of an ongoing study.
